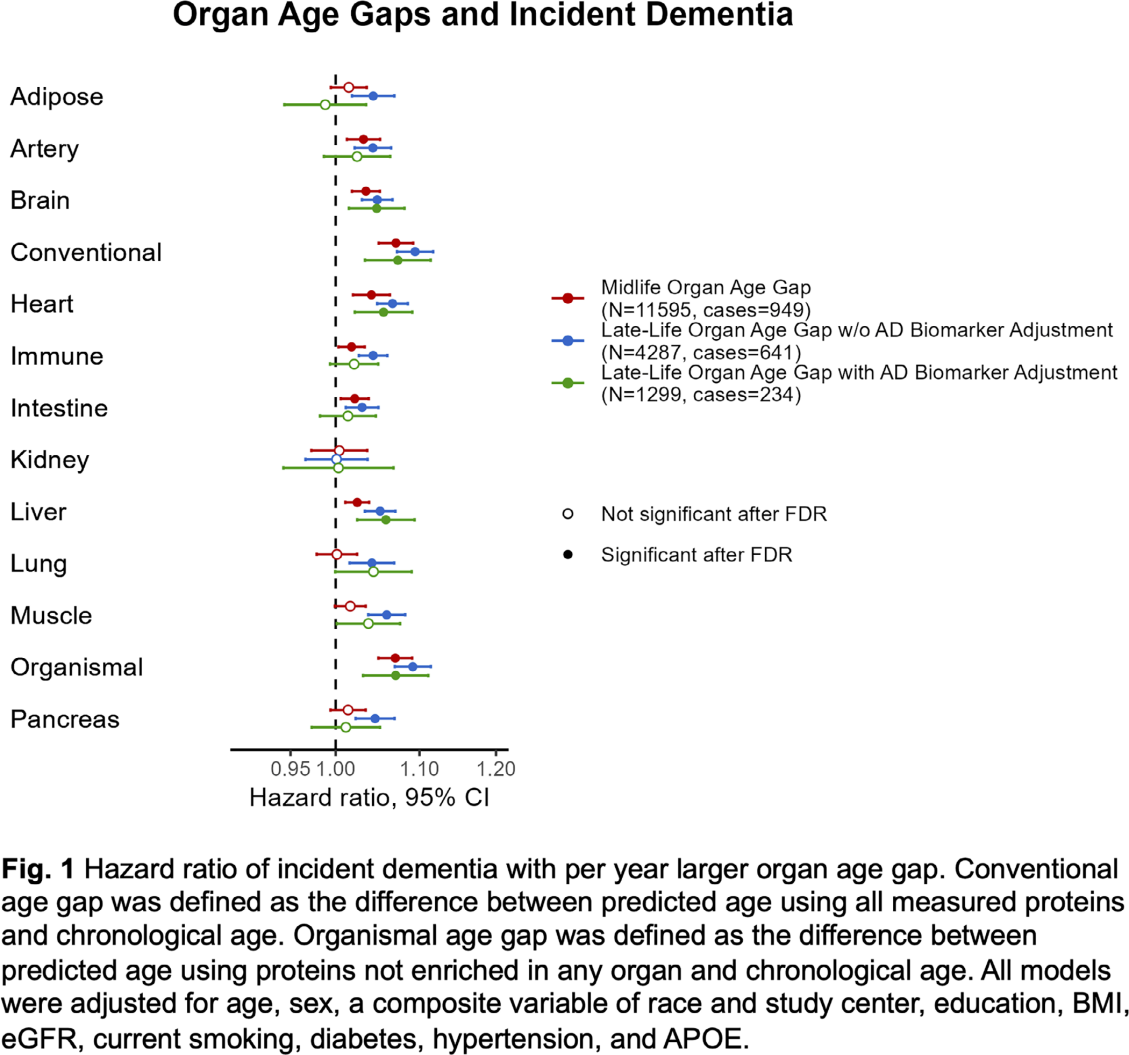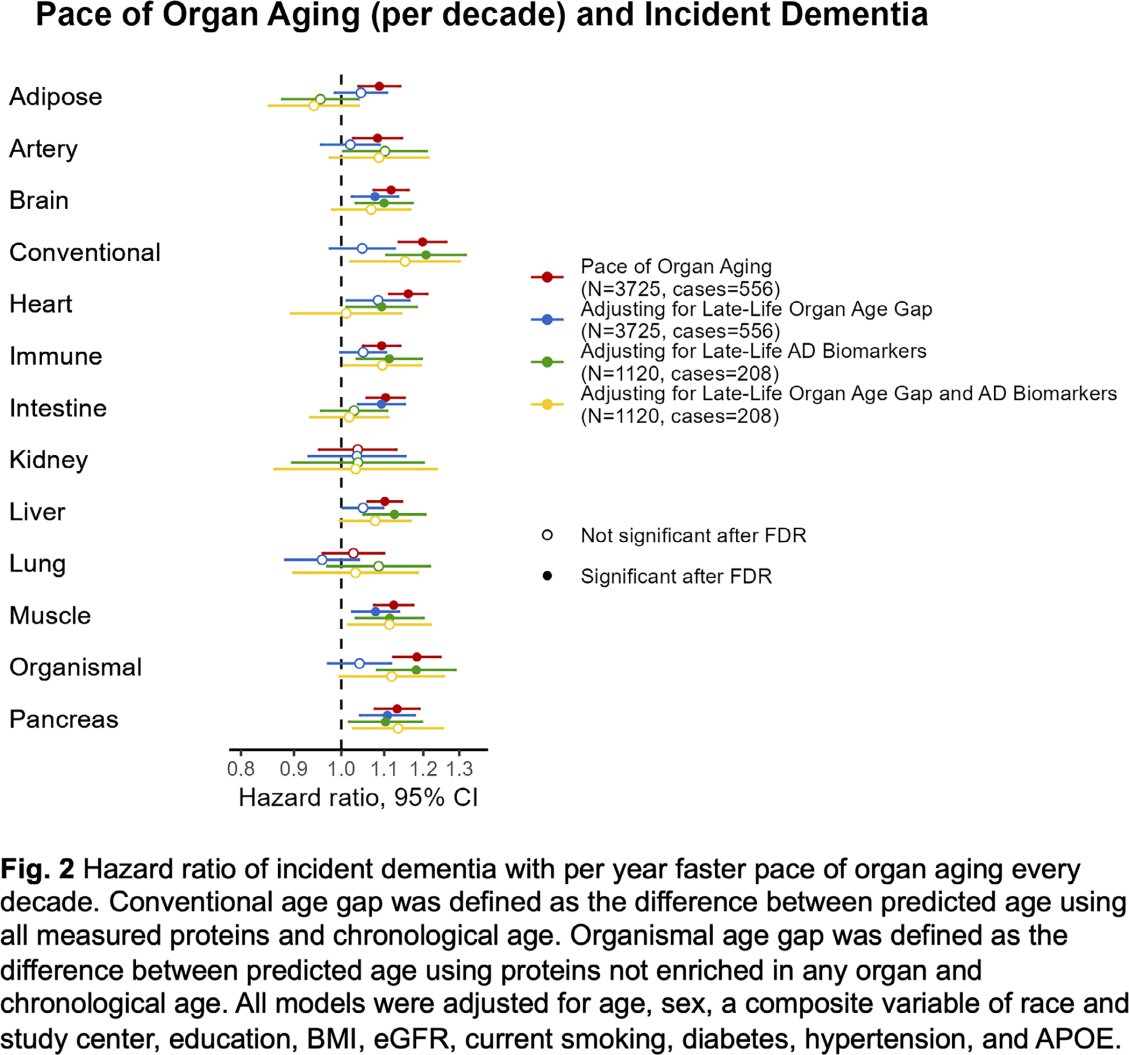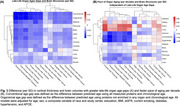# Plasma protein‐based organ age gap and pace of organ aging were associated with incident dementia and brain structures

**DOI:** 10.1002/alz70856_100937

**Published:** 2025-12-25

**Authors:** Fangyu Liu, Aditya Surapaneni, Jingsha Chen, Cassandra M Joynes, Ramon Casanova, Wenyu Zhou, Basilio Cieza, Jarod Rutledge, Hamilton Se‐Hwee Oh, Alex Boches, Tony Wyss‐Coray, Gabriela T Gomez, Michael R Duggan, Anna Prizment, Sanaz Sedaghat, Michael E. Griswold, Shuo Wang, Josef Coresh, Morgan E Grams, Keenan A. Walker

**Affiliations:** ^1^ Laboratory of Behavioral Neuroscience, National Institute on Aging, Intramural Research Program, Baltimore, MD, USA; ^2^ New York University, New York, NY, USA; ^3^ Department of Epidemiology, Johns Hopkins University Bloomberg School of Public Health, Baltimore, MD, USA; ^4^ Wake Forest University School of Medicine, Winston‐Salem, NC, USA; ^5^ Teal Omics, Palo Alto, CA, USA; ^6^ Columbia University, New York, NY, USA; ^7^ Wu Tsai Neurosciences Institute, Stanford University, Stanford, CA, USA; ^8^ Brigham and Women's Hospital, Boston, MA, USA; ^9^ University of Minnesota, Minneapolis, MN, USA; ^10^ University of Mississippi Medical Center, The MIND Center, Jackson, MS, USA

## Abstract

**Background:**

Organs may age at different rates. We used a plasma protein‐based algorithm that captures organ age to investigate how older age in different organs compared to chronological age (henceforth, organ age gaps) and greater longitudinal increase in organ age gaps (henceforth, pace of organ aging) influence dementia risk and brain structures.

**Method:**

Using the Atherosclerosis Risk in Communities (ARIC) study, we generated 11 organ age gaps in midlife (*N* = 11,595, mean age 57±6 years) and in late life (*N* = 4,287, mean age 75±5 years). Paces of organ aging were standardized by decades between midlife and late life (*N* = 3,725). We used Cox proportional hazard models to relate (i) midlife organ age gaps to 20‐year dementia risk, (ii) late‐life organ age gaps to 7‐year dementia risk, and (iii) paces of organ aging to 7‐year dementia risk. Linear regression models were used to relate late‐life organ age gaps and paces of organ aging to concurrent MRI‐defined brain volume and cortical thickness (standardized by SD). All models were adjusted for demographic factors, BMI, eGFR, smoking status, APOE, diabetes, and hypertension.

**Result:**

Larger midlife age gaps in artery, brain, heart, immune, intestine, and liver (HR=1.02‐1.07, q<0.05) and larger late‐life age gaps in all organs except kidney (HR=1.03‐1.09, q<0.05) were associated with higher dementia risk (Figure 1). Late‐life brain, heart, and liver age gaps remained significant after further adjustment of Aß42:Aß40 ratio, GFAP, NfL, and *p*‐tau181. Faster paces of aging in brain, heart, intestine, muscle, and pancreas were associated with higher dementia risk independent of their respective late‐life organ age gaps (HR=1.04‐1.05, q<0.05, Figure 2). Larger late‐life age gaps in all organs except kidney were also associated with reduced cortical thickness and/or volumes (ß=‐0.01 to ‐0.04 SD, q<0.05, Figure 3A). Independent of their respective late‐life age gaps, faster pace of immune and pancreas aging was associated with lower total cortical thickness (ß=‐0.02 SD, q<0.05) and temporal‐parietal volume (ß=‐0.02, q<0.05, Figure 3B), respectively.

**Conclusion:**

Older age gaps and faster pace of aging in organs other than the brain, e.g., heart, immune system, and pancreas, may play an important role in dementia risk and structural brain changes.